# Supplementary material for: Protein design of two-component tubular assemblies similar to cytoskeletons
Source: Nat Commun. 2025 Jul 22;16:6738. doi: 10.1038/s41467-025-62076-3 (PMC12283931; doi:10.1038/s41467-025-62076-3)
Supplement: Supplementary file 1 — Supplementary Information [file 41467_2025_62076_MOESM1_ESM.pdf]

Supplementary Figures

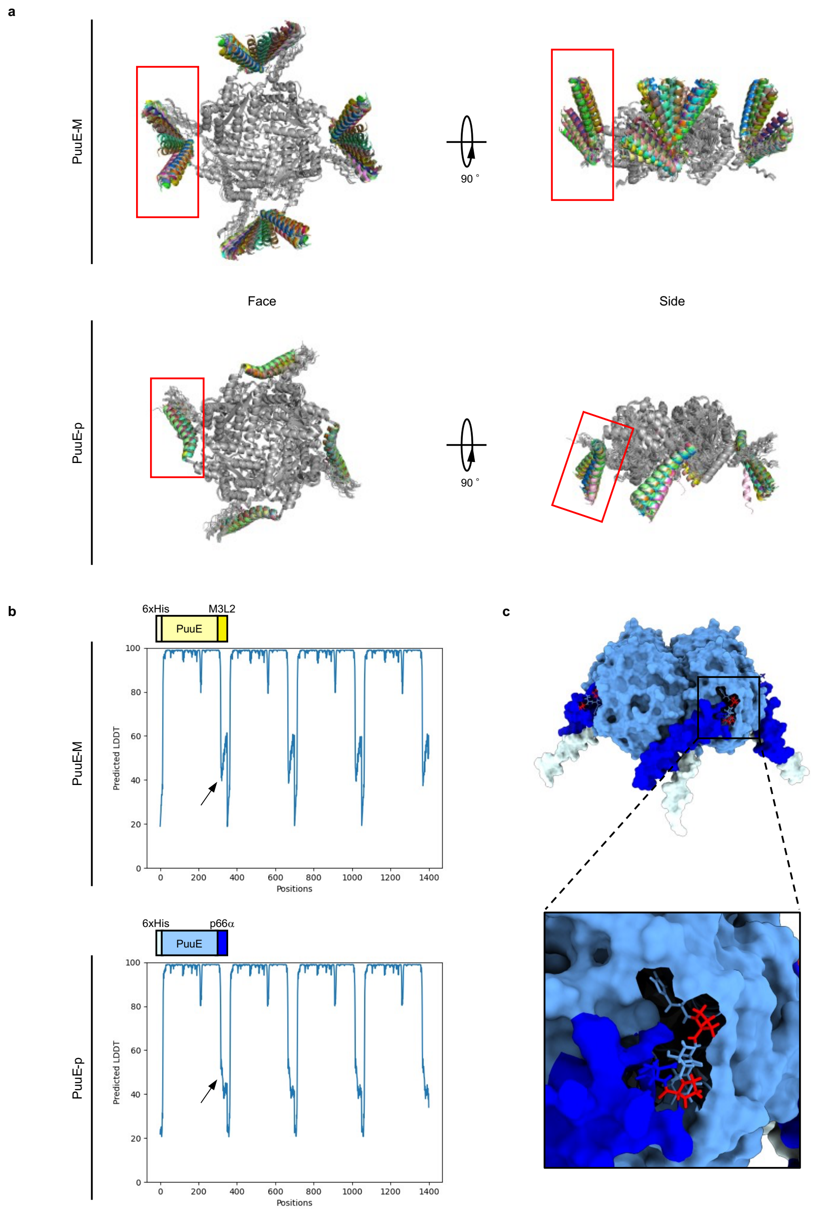

Supplementary Fig. 1. AF2 prediction of PuuE-M and PuuE-p. | a, Fifty prediction models overlapped for PuuE-M (top) and PuuE-p (bottom). Peptide parts, M3L2 and p66α, are indicated with a red box. **b,** Predicted local distance difference test plots for the most reliable prediction models for PuuE-M (top) and PuuE-p (bottom). Arrows indicate the N-terminal region of M3L2 and p66α. For these regions, PuuE-M has a lower predictive reliability than does PuuE-p, suggesting that the structure may be more flexible. **c,** The most reliable prediction model for PuuE-p. The region from the C-terminus of PuuE to the N-terminus of p66α (i.e. ^{313}HPYTPE^{318}) is depicted by a stick model. The two Pro residues highlighted in red are thought to be responsible for the rigidity of the PuuE-p structure. Because of the rigidity of PuuE-p, the final product of the mixture was predicted to be a closed structure, as shown in **Fig. 1d**.

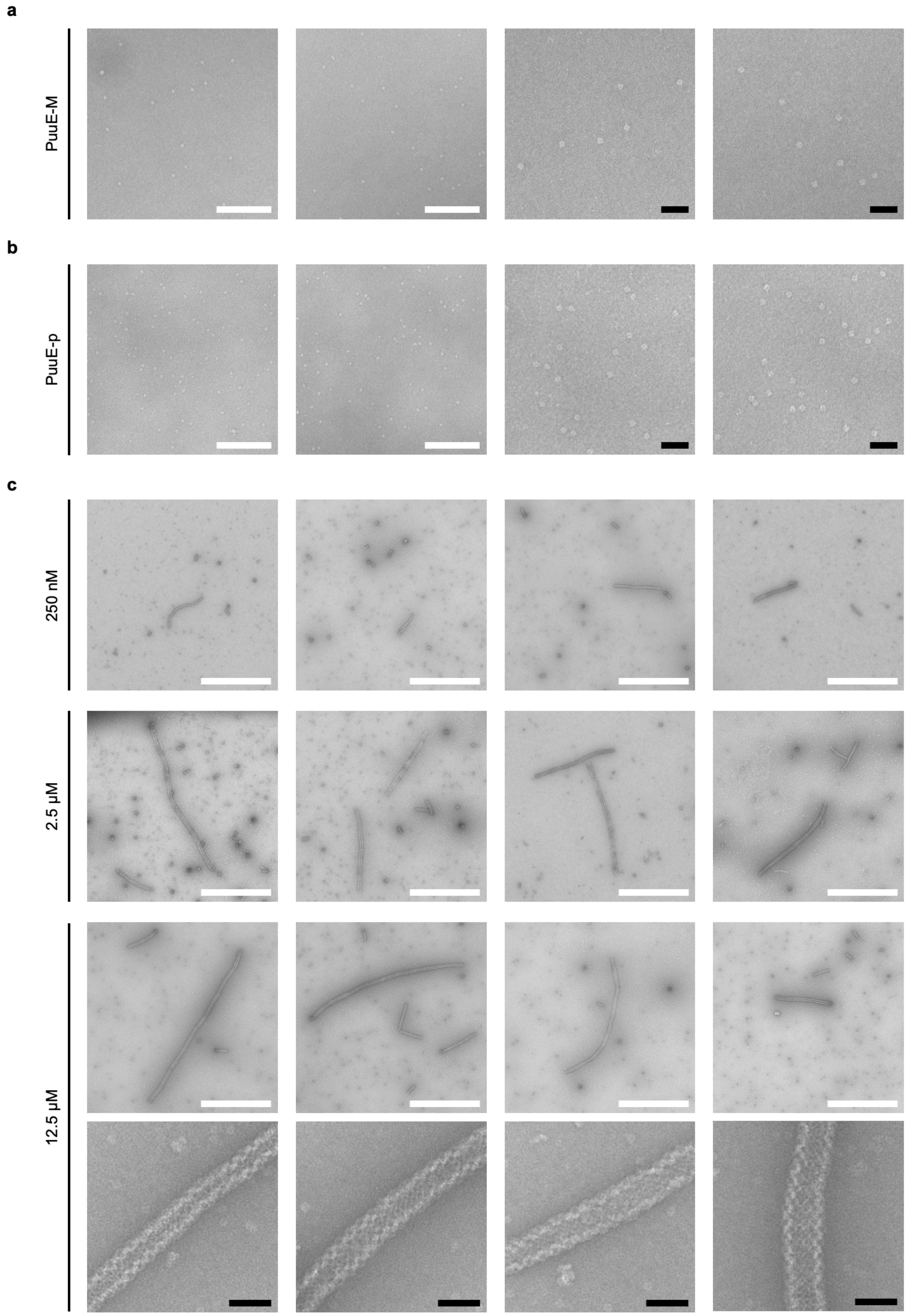

Supplementary Fig. 2. nsTEM characterisation of PuuE-M, PuuE-p, and the mixture for PuuE-M and PuuE-p. | a, 12.5 μM PuuE-M or **b,** 12.5 μM PuuE-p in NaCl (+) buffer was incubated at 40 °C for 24 h. Scale bars, 200 nm (white), 50 nm (black). **c,** Dependency of PuuE tube assemblies on protein concentration. 250 nM (top), 2.5 μM (middle), and 12.5 μM (bottom) of PuuE-M and PuuE-p each in NaCl (+) buffer was incubated at 40 °C for 24 h and imaged by nsTEM. The tube structure observed in the nsTEM images was flexible as it was curved and collapsed. Scale bars, 1 μm (white), 50 nm (black).

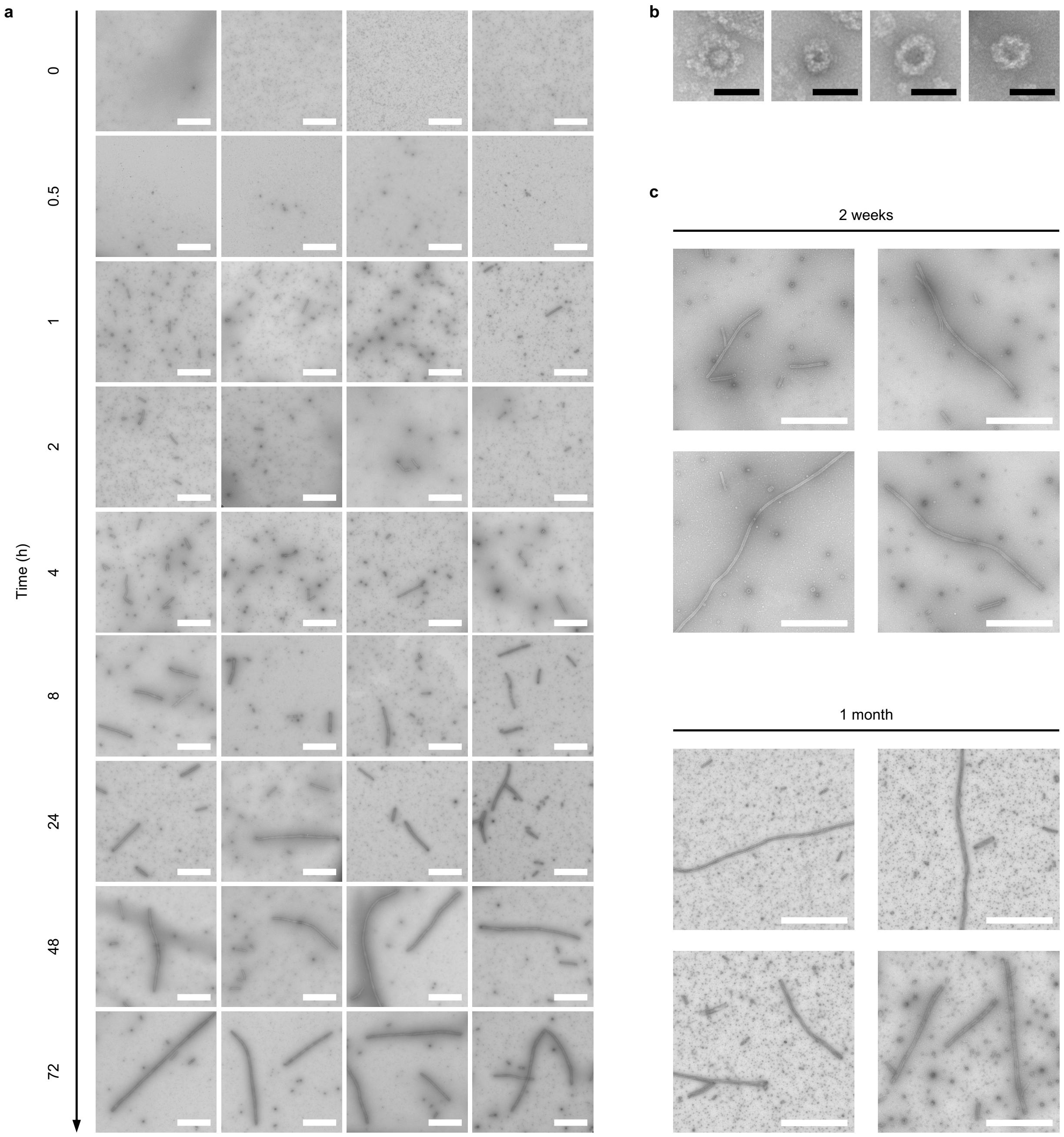

Supplementary Fig. 3. Time dependence of PuuE tube assemblies and their stability over time. | a, 12.5 μM of PuuE-M and PuuE-p each in NaCl (+) buffer was incubated at 40 °C for indicated time points and imaged via nsTEM. **b,** Ring-like assemblies seen in addition to tube structures during the observation of the samples in **a**. **c,** After 24 h of tube formation, the sample was kept at 25 ± 1 °C for the indicated time and imaged using nsTEM. Tube structures remained unchanged after 2 weeks and even after 1 month, suggesting stability. Scale bars, 1 μm (white), 50 nm (black).

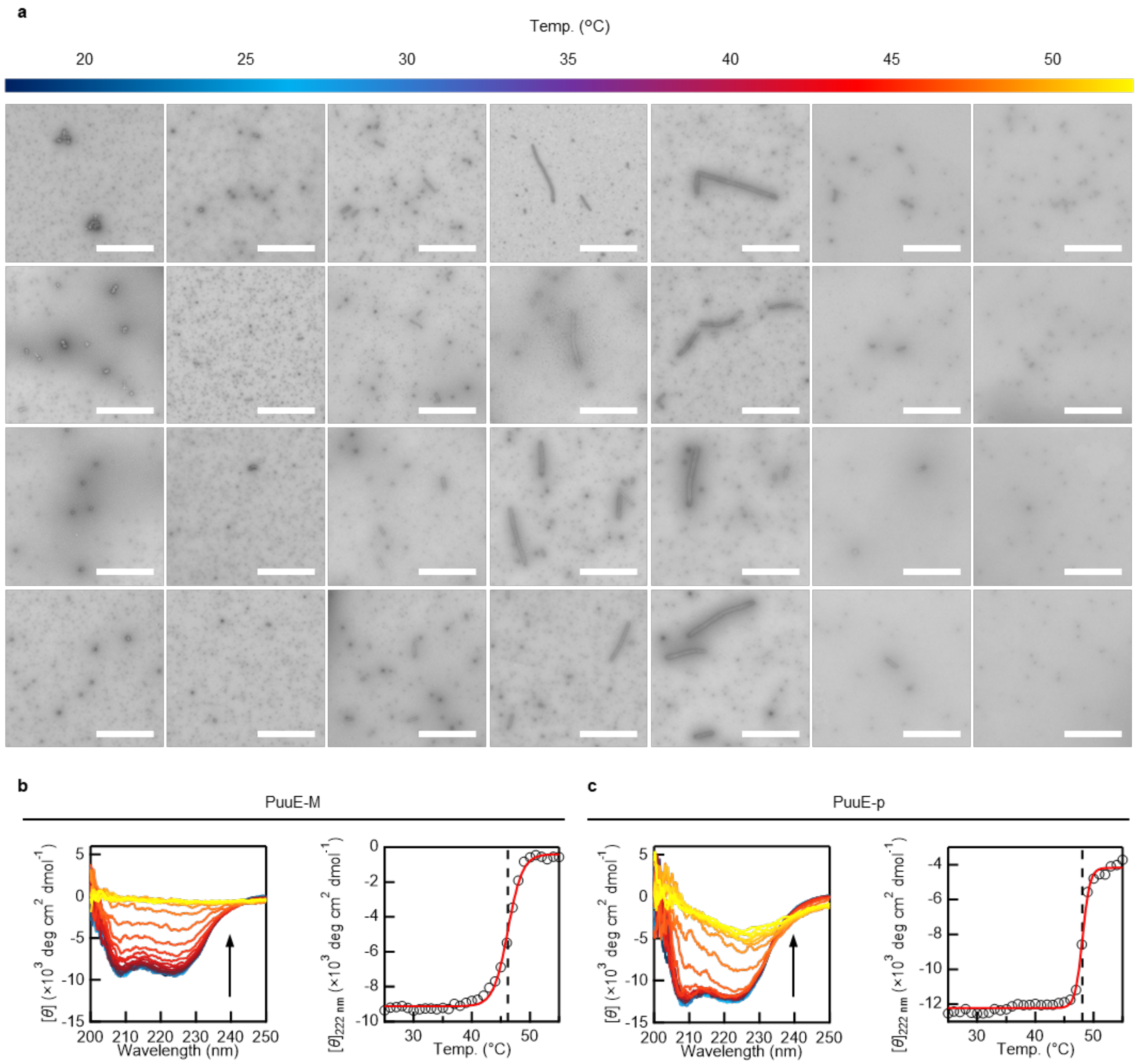

Supplementary Fig. 4. Temperature dependence of PuuE tube assemblies and determination of T_m for PuuE-M and PuuE-p. | a, 12.5 µM of PuuE-M and PuuE-p each in NaCl (+) buffer was incubated at the indicated temperature for 24 h and imaged via nsTEM. Scale bars, 1 µm. **b, c,** T_m measurements using CD for PuuE-M (**b**) and PuuE-p (**c**). 2.5 µM of PuuE-M or PuuE-p in NaCl (+) buffer was incubated from 25 to 55 °C with temperature change of 1 °C/min. Left panel, overall CD spectra; right panel, thermal denaturation profiles. Source data are provided as a Source Data file.

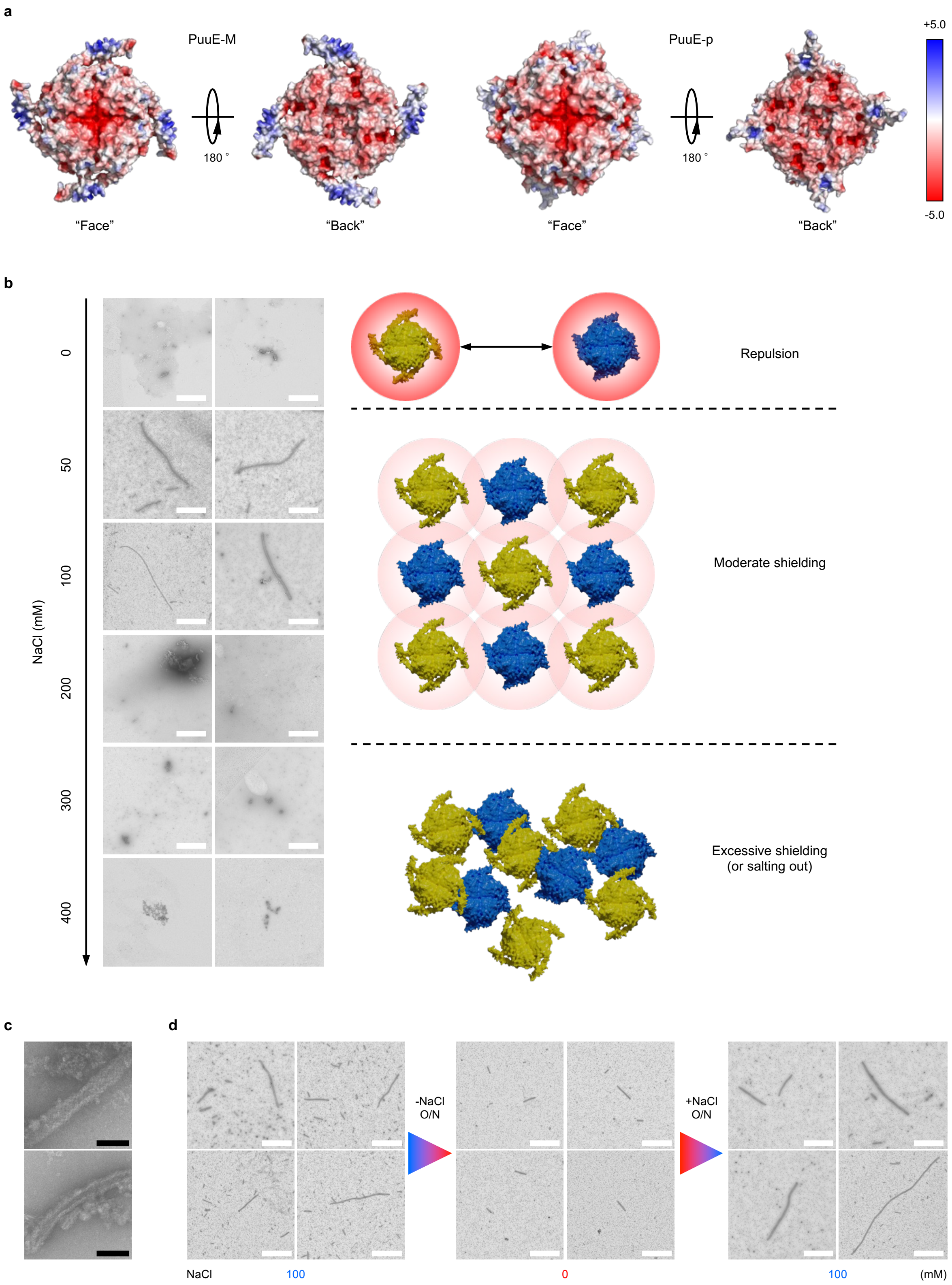

a
PuuE-M
180 °
"Face"
"Back"
PuuE-p
180 °
"Face"
"Back"
+5.0
-5.0
b
NaCl (mM)
0
50
100
200
300
400
Repulsion
Moderate shielding
Excessive shielding
(or salting out)
c
d
-NaCl
O/N
+NaCl
O/N
NaCl
100
0
100
(mM)

Supplementary Fig. 5. Salt concentration dependence and reversibility of PuuE tube assemblies. | a, Surface electrostatic potential calculation for PuuE-M (left) and PuuE-p (right) at pH 8.0. **b,** Left, 12.5 μM of PuuE-M and PuuE-p each in NaCl (+) buffer was incubated at 40 °C for 24 h with indicated NaCl concentration and imaged via nsTEM. Right, diagram of salt concentration effects described in the main text. **c,** 6.25 μM of His-tag cleaved PuuE-M and His-tag cleaved PuuE-p each in 400 mM NaCl buffer was incubated at 40 °C for 24 h and imaged via nsTEM. **d,** Additional images in **Fig. 2d** prove the reversibility of tubular assemblies. These images were used for statistical analysis of tube length, as shown in **Fig. 2e**. Scale bars, 1 μm (white), 100 nm (black).

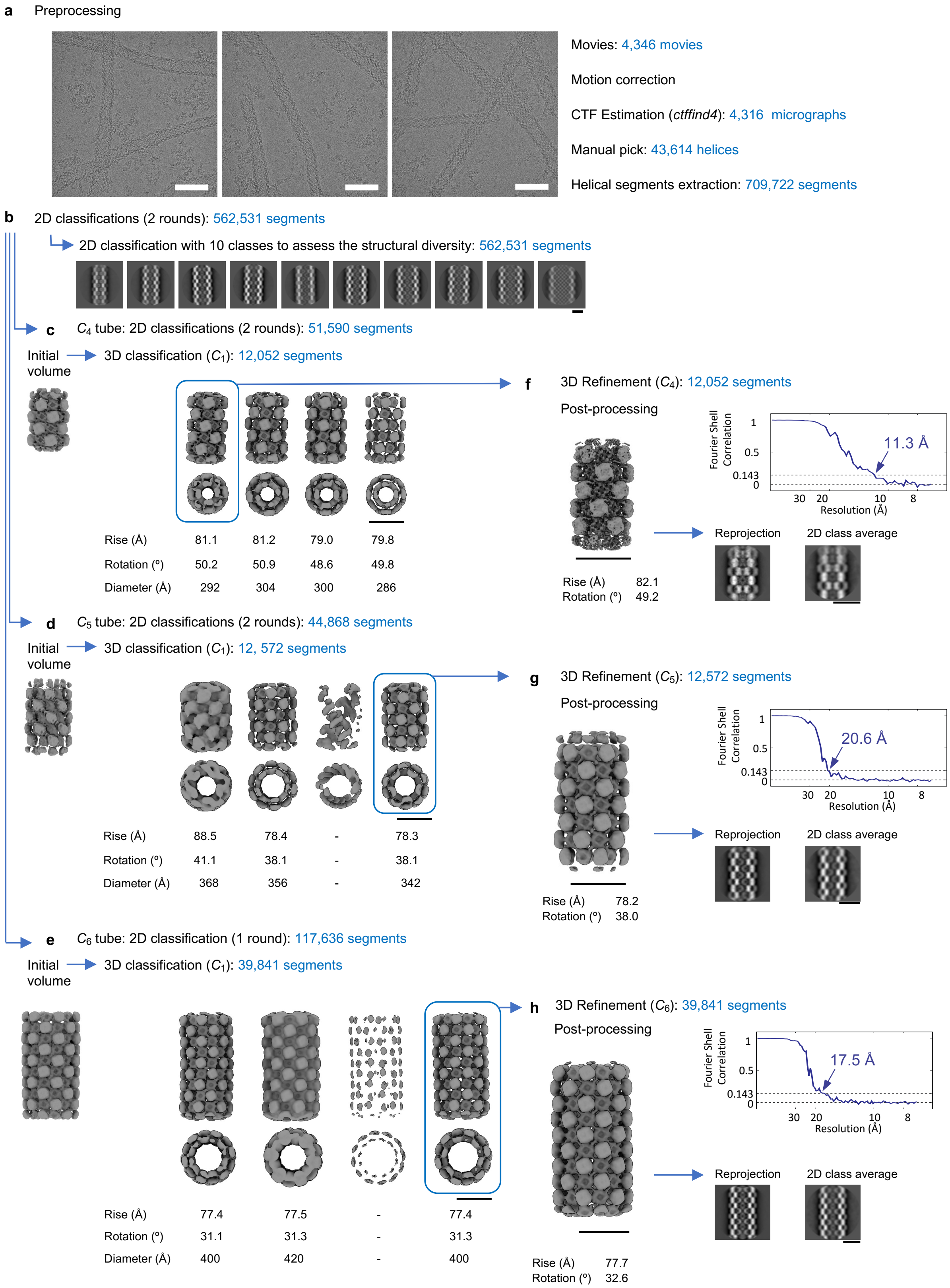
a
Preprocessing
Movies: 4,346 movies
Motion correction
CTF Estimation (ctffind4): 4,316 micrographs
Manual pick: 43,614 helices
Helical segments extraction: 709,722 segments
b
2D classifications (2 rounds): 562,531 segments
2D classification with 10 classes to assess the structural diversity: 562,531 segments
c
C_4 tube: 2D classifications (2 rounds): 51,590 segments
Initial volume
3D classification (C_1): 12,052 segments
Rise (Å) 81.1 81.2 79.0 79.8
Rotation (°) 50.2 50.9 48.6 49.8
Diameter (Å) 292 304 300 286
f
3D Refinement (C_4): 12,052 segments
Post-processing
Fourier Shell Correlation
1
0.5
0.143
0
30 20 10 8
Resolution (Å)
11.3 Å
Reprojection
2D class average
Rise (Å) 82.1
Rotation (°) 49.2
d
C_5 tube: 2D classifications (2 rounds): 44,868 segments
Initial volume
3D classification (C_1): 12, 572 segments
Rise (Å) 88.5 78.4 - 78.3
Rotation (°) 41.1 38.1 - 38.1
Diameter (Å) 368 356 - 342
g
3D Refinement (C_5): 12,572 segments
Post-processing
Fourier Shell Correlation
1
0.5
0.143
0
30 20 10 8
Resolution (Å)
20.6 Å
Reprojection
2D class average
Rise (Å) 78.2
Rotation (°) 38.0
e
C_6 tube: 2D classification (1 round): 117,636 segments
Initial volume
3D classification (C_1): 39,841 segments
Rise (Å) 77.4 77.5 - 77.4
Rotation (°) 31.1 31.3 - 31.3
Diameter (Å) 400 420 - 400
h
3D Refinement (C_6): 39,841 segments
Post-processing
Fourier Shell Correlation
1
0.5
0.143
0
30 20 10 8
Resolution (Å)
17.5 Å
Reprojection
2D class average
Rise (Å) 77.7
Rotation (°) 32.6

Supplementary Fig. 6. Cryo-EM image processing workflow of the PuuE tubes. | a, Representative motion-corrected micrographs (left panels) and preprocessing steps, with numbers of micrographs and extracted segments indicated. **b,** 2D classification revealing tubes of varying diameters. **c, d, e,** Subsets of C_4, C_5, and C_6 tubes subjected to additional 2D and 3D classifications into four classes using an initial 3D volume from cryoSPARC. Side (upper panels) and top (bottom panels) views of the 3D class averages are shown for each class, with corresponding helical parameters (rise, rotation, and diameter). The 3D class with the most detailed features was selected for further refinement. **f, g, h,** Final 3D reconstructions with refined helical parameters (left), FSC plots for a global resolution estimation at the 0.143 threshold (top right), and comparison of 2D reprojections with 2D class averages for structural validation (bottom right). Scale bars: 100 nm (white), 250 Å (black).

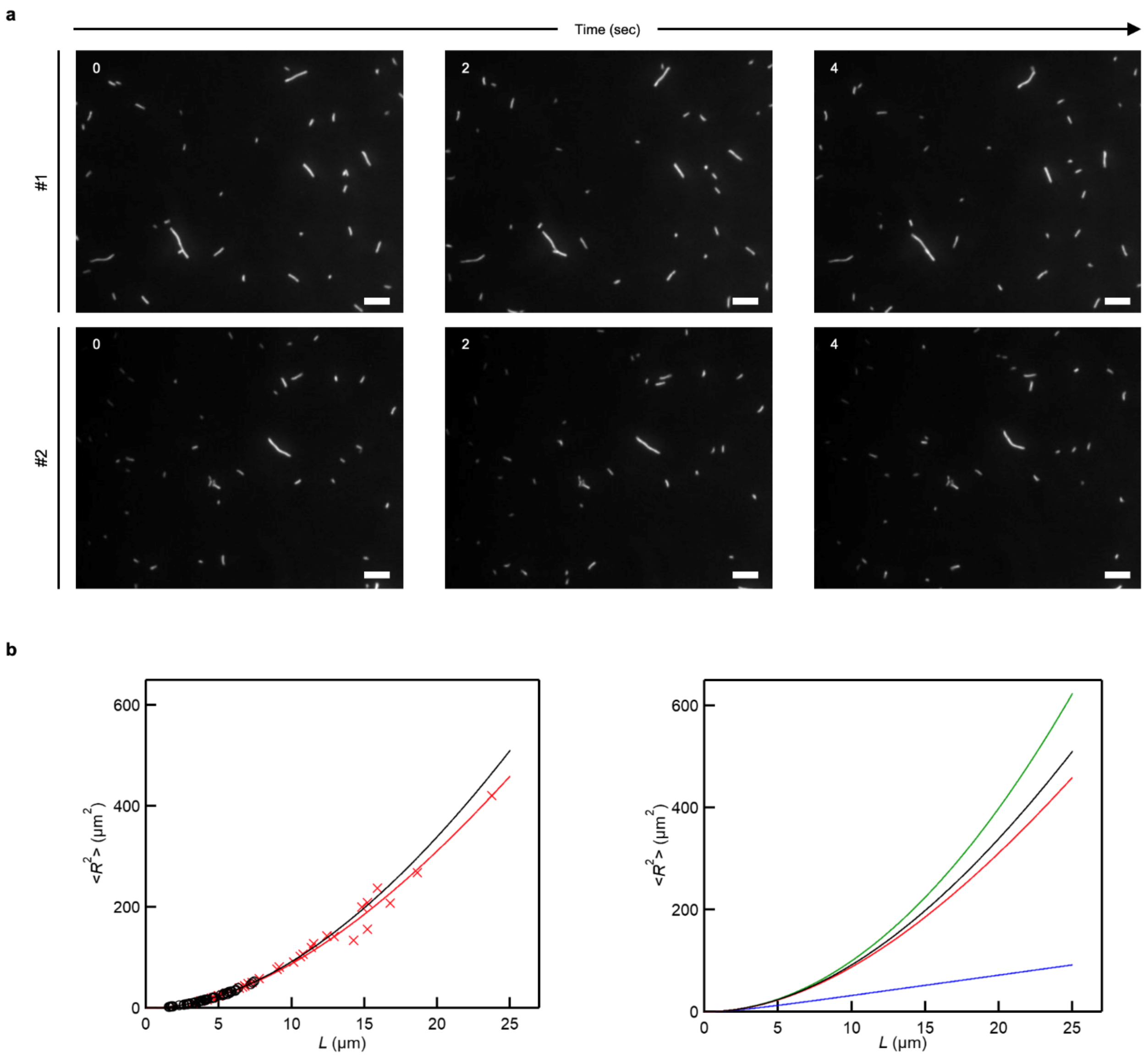

Supplementary Fig. 7. Dynamic flexibility and persistence length analysis of PuuE tube. | a, Time-lapse images of random bending of tube structures observed using TIRFM. Snapshots at the starting point (0 s) and after 2 and 4 s. Scale bars, 5 µm. **b,** Left, relationship between contour length (L) and mean square of end-to-end distance of the tube structures ($<R^2>$). The continuous lines represent curves (black for PuuE tube, red for actin filament) fitted to the experimental data (black open circle for PuuE tube, red cross mark for actin filament). A wider range of L values than that in **Fig. 3d** is shown. Right, the theoretical curve of microtubules (green line, L_p = 10 mm[41-43]) and that of intermediate filaments (blue line, L_p = 1 µm[44]) are overlaid on PuuE tubes (black) and actin filaments (red). As L increases, the fitted curve of the tubes (black) increases similar to that of the actin filament but increases more slowly than the microtubule's curve and faster than that of the intermediate filament. Source data are provided as a Source Data file.

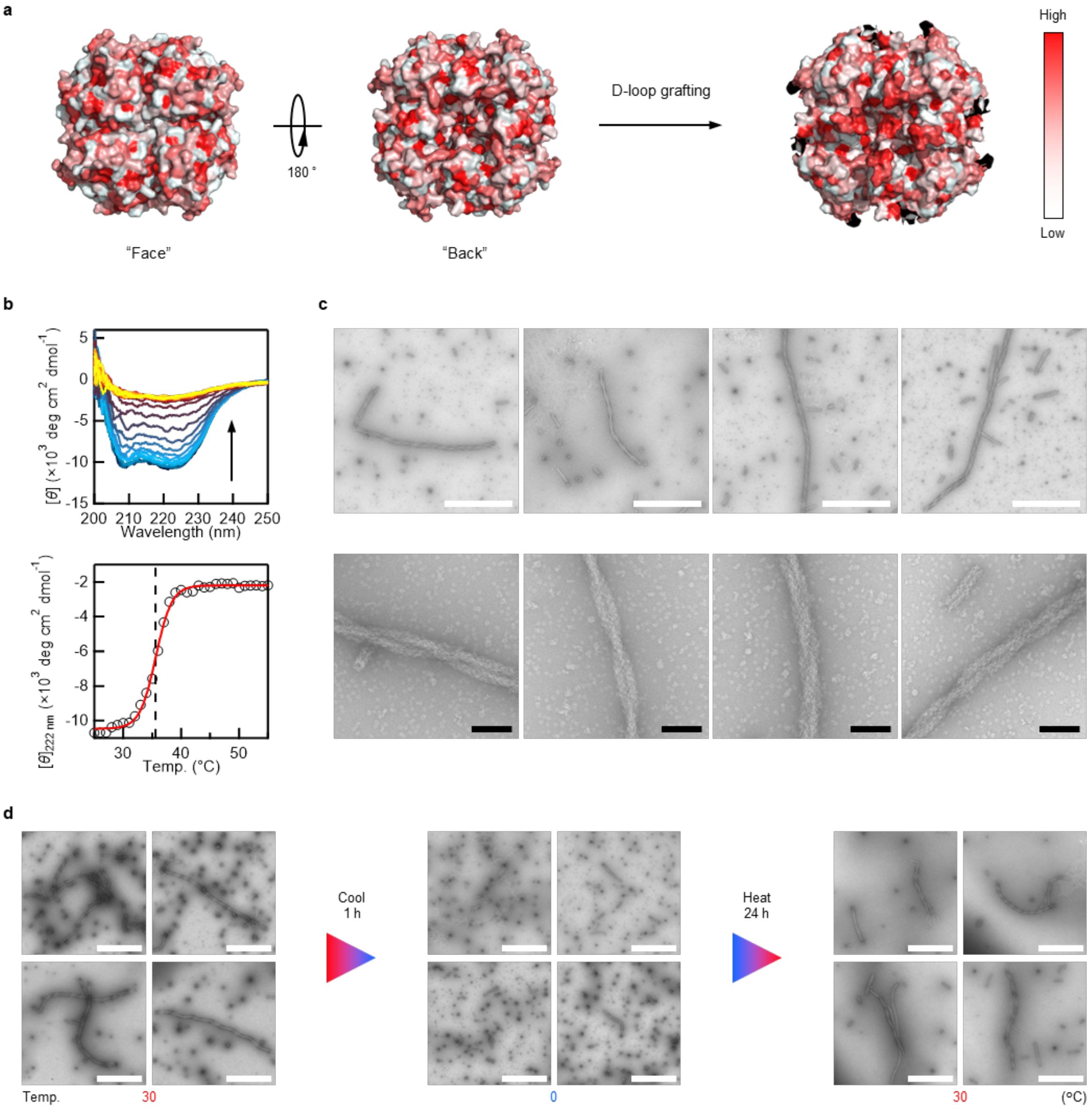

Supplementary Fig. 8. D-loop grafting to emulate actin filaments. | **a,** Surface hydrophobicity calculation for PuuE. D-loop was grafted into the 'back' side of PuuE owing to the hydrophobic nature of a prominent indentation. **b,** T_m measurement of PuuE(D-loop)-M via CD. 2.5 μM of PuuE(D-loop)-M in NaCl (+) buffer was incubated from 25 to 55 °C with temperature change of 1 °C/min. Top: overall CD spectra; bottom: thermal denaturation profiles, respectively. **c,** 12.5 μM of PuuE(D-loop)-M and PuuE-p in NaCl (+) buffer were incubated at 30 °C for 24 h and imaged using nsTEM. A novel helical pattern of two or three intertwined tubes was clearly observed. Flexibility was also noted when curved structures were observed. **d,** Additional images for reversibility of tube formation depends on temperature changes in **Fig. 4c**. For this observation, we focused on the presence of tube structures with helical conformations. After 1

h at 0 °C, there were no such structures observed via nsTEM. Scale bars, 1 μm (white), 100 nm (black). Source data are provided as a Source Data file.

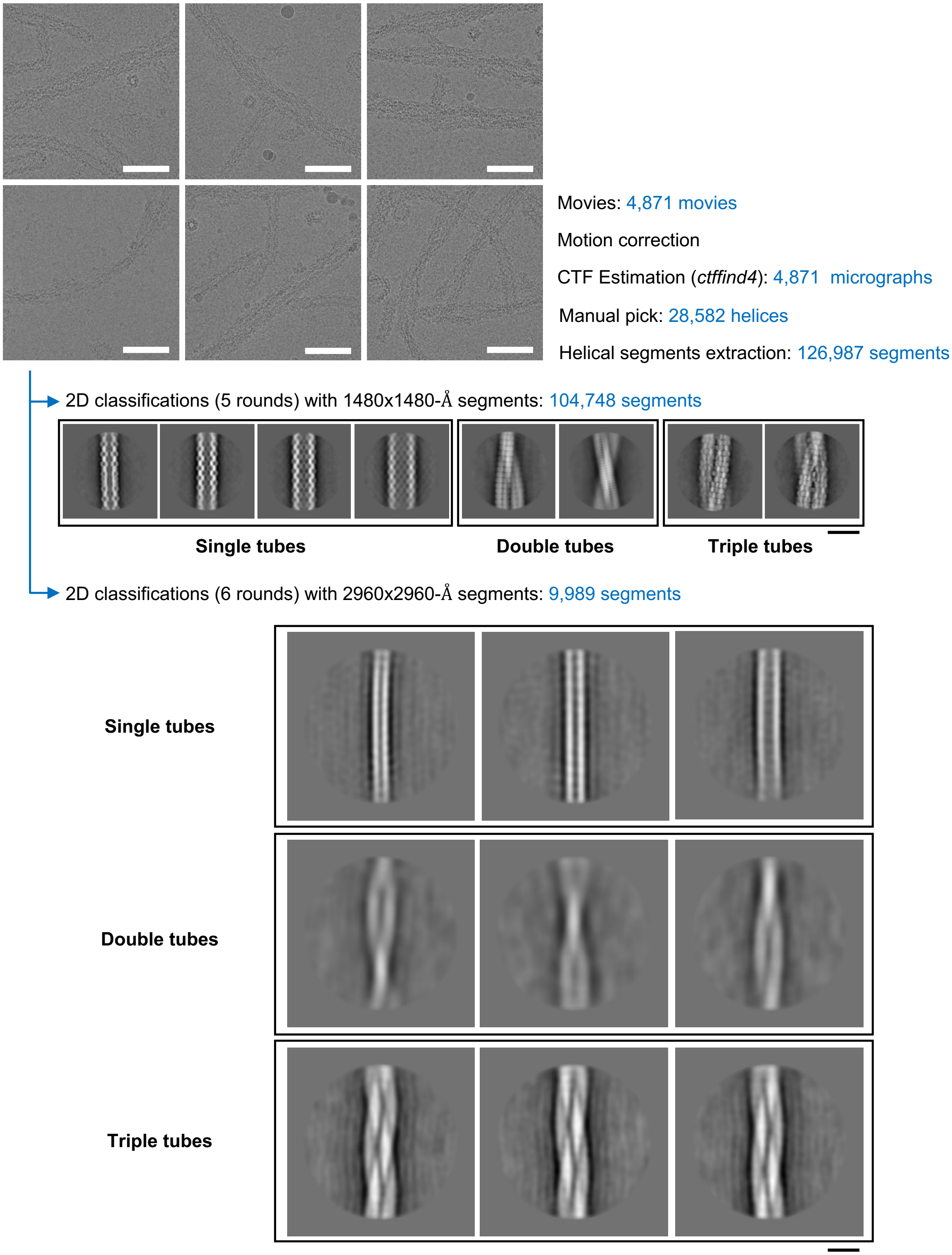

Supplementary Fig. 9. Cryo-EM image processing workflow of PuuE D-loop tubes prepared at 25 ± 1 °C. | Flowchart illustrating the image processing steps. Scale bars: 100 nm (white), 500 Å (black).

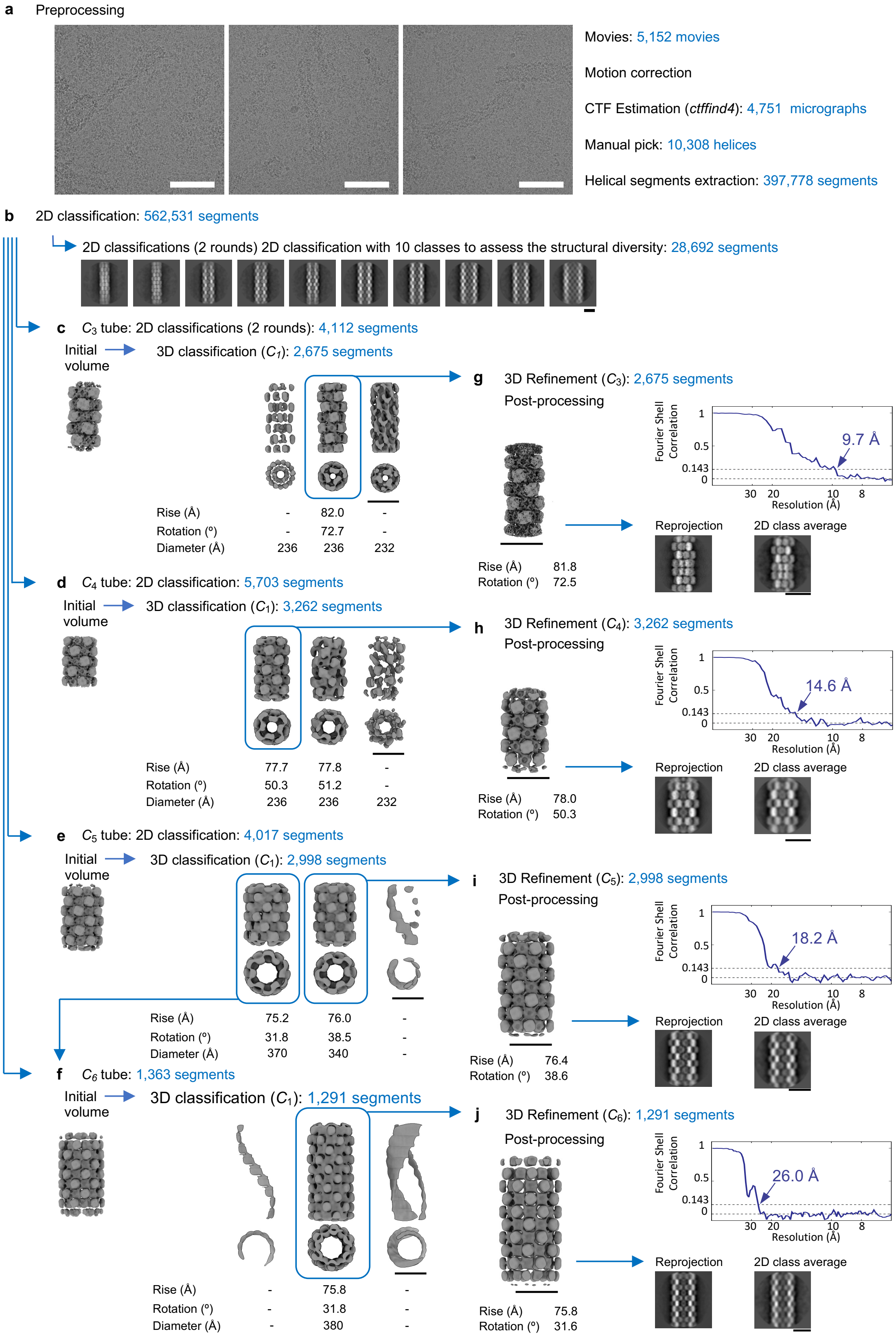

a
Preprocessing
Movies: 5,152 movies
Motion correction
CTF Estimation (ctffind4): 4,751 micrographs
Manual pick: 10,308 helices
Helical segments extraction: 397,778 segments
b
2D classification: 562,531 segments
2D classifications (2 rounds) 2D classification with 10 classes to assess the structural diversity: 28,692 segments
c
C_3 tube: 2D classifications (2 rounds): 4,112 segments
Initial volume
3D classification (C_1): 2,675 segments
Rise (Å) - 82.0 -
Rotation (°) - 72.7 -
Diameter (Å) 236 236 232
g
3D Refinement (C_3): 2,675 segments
Post-processing
Fourier Shell Correlation
1
0.5
0.143
0
9.7 Å
30 20 10 8
Resolution (Å)
Rise (Å) 81.8
Rotation (°) 72.5
Reprojection
2D class average
d
C_4 tube: 2D classification: 5,703 segments
Initial volume
3D classification (C_1): 3,262 segments
Rise (Å) 77.7 77.8 -
Rotation (°) 50.3 51.2 -
Diameter (Å) 236 236 232
h
3D Refinement (C_4): 3,262 segments
Post-processing
14.6 Å
Rise (Å) 78.0
Rotation (°) 50.3
Reprojection
2D class average
e
C_5 tube: 2D classification: 4,017 segments
Initial volume
3D classification (C_1): 2,998 segments
Rise (Å) 75.2 76.0 -
Rotation (°) 31.8 38.5 -
Diameter (Å) 370 340 -
i
3D Refinement (C_5): 2,998 segments
Post-processing
18.2 Å
Rise (Å) 76.4
Rotation (°) 38.6
Reprojection
2D class average
f
C_6 tube: 1,363 segments
Initial volume
3D classification (C_1): 1,291 segments
Rise (Å) - 75.8 -
Rotation (°) - 31.8 -
Diameter (Å) - 380 -
j
3D Refinement (C_6): 1,291 segments
Post-processing
26.0 Å
Rise (Å) 75.8
Rotation (°) 31.6
Reprojection
2D class average

Supplementary Fig. 10. Cryo-EM image processing workflow of PuuE D-loop tubes preincubated on ice to unwind the helical structures. | a, Representative motion-corrected micrographs (left) and preprocessing steps, with numbers of micrographs and extracted segments indicated. **b,** 2D classification revealing tubes of varying diameters. **c, d, e, f,** Subsets from 2D class (**b**) showing C_3, C_4, C_5, and C_6 tubes subjected to additional 2D and 3D classifications into three classes using an initial 3D volume from cryoSPARC. Side (upper panels) and top (bottom panels) views of the 3D class averages are shown for each class, with corresponding helical parameters (rise, rotation, and diameter). The 3D class with the most detailed features was selected for further refinement. The C_5 tube subset from 2D classification (**e**) was later found to contain C_6 tubes as well during 3D classification. These segments were merged with the C_6 tube subset from 2D classification (**f**). **g, h, i, j,** Final 3D reconstructions with refined helical parameters (left), FSC plots for a global resolution estimation at the 0.143 thresholds (top right), and comparison of 2D reprojections with 2D class averages for structural validation (bottom right). Scale bars: 100 nm (white), 250 Å (black).

Supplementary Tables

PuuE-M; 6xHis-TEVcs-PuuE-M3L2
MHHHHHHENLYFQGVDYPRDLIGYGSNPPHPHWPGKARIALSFVLNYEEGGERNILHGDKESEA
FLSEMVSAQPLQGERNMSMESLYEYGSRAGVWRILKLFKAFDIPLTIFAVAMAAQRHPDVIRAMV
AAGHEICSHGYRWIDYQYMDEAQEREHMLEAIRILTELTGERPLGWYTGRTGPNTRRLVMEEGG
FLYDCDTYDDDLPYWEPNNPTGKPHLVIPYTLDTNDMRFTQVQGFNKGDDFFEYLKDAFDVLYA
EGAEAPKMLSIGLHCRLIGRPARLAALQRFIEYAKSHEQVWFTRRVDIARHWHATHPYTSGQLVT
PADIRRQARRVKKARERLAKALQADRLA

PuuE-p; 6xHis-TEVcs-PuuE-p66α
MHHHHHHENLYFQGVDYPRDLIGYGSNPPHPHWPGKARIALSFVLNYEEGGERNILHGDKESEA
FLSEMVSAQPLQGERNMSMESLYEYGSRAGVWRILKLFKAFDIPLTIFAVAMAAQRHPDVIRAMV
AAGHEICSHGYRWIDYQYMDEAQEREHMLEAIRILTELTGERPLGWYTGRTGPNTRRLVMEEGG
FLYDCDTYDDDLPYWEPNNPTGKPHLVIPYTLDTNDMRFTQVQGFNKGDDFFEYLKDAFDVLYA
EGAEAPKMLSIGLHCRLIGRPARLAALQRFIEYAKSHEQVWFTRRVDIARHWHATHPYTPEERER
MIKQLKEELRLEEAKLVLLKKLRQSQIQ

PuuE(D-loop)-M; 6xHis-TEVcs-PuuE(D-loop)-M3L2
MHHHHHHENLYFQGVDYPRDLIGYGSNPPHPHWPGKARIALSFVLNYEEGGERNILHGDKESEA
FLSEMVSAQPLQQGVMVGMGQKGERNMSMESLYEYGSRAGVWRILKLFKAFDIPLTIFAVAMAA
QRHPDVIRAMVAAGHEICSHGYRWIDYQYMDEAQEREHMLEAIRILTELTGERPLGWYTGRTGP
NTRRLVMEEGGFLYDCDTYDDDLPYWEPNNPTGKPHLVIPYTLDTNDMRFTQVQGFNKGDDFF
EYLKDAFDVLYAEGAEAPKMLSIGLHCRLIGRPARLAALQRFIEYAKSHEQVWFTRRVDIARHWH
ATHPYTSGQLVTPADIRRQARRVKKARERLAKALQADRLA

Supplementary Table 1. Amino Acid sequences of PuuE-M, PuuE-p, and PuuE(D-loop)-M.

PuuE tube

	#1 C_4 tube (EMD-60617)	#2 C_5 tube (EMD-60618)	#3 C_6 tube (EMD-60619)
**Data collection and processing**			
Magnification	120,000		
Voltage (kV)	200		
Electron exposure ($e^-/Å^2$)	40		
Defocus range (µm)	–0.8 to –1.6		
Pixel size (Å)	1.22		
Symmetry imposed	C_4 helical	C_5 helical	C_6 helical
Initial helical segments (no.)	709,722	709,722	709,722
Final helical segments (no.)	12,052	12,572	39,841
Map resolution (Å)	11.3	20.6	17.5
FSC threshold	0.143	0.143	0.143

2D analysis of the PuuE D-loop tube prepared at 25 ± 1 °C

	#1 Tubes
**Data collection and processing**	
Magnification	150,000
Voltage (kV)	200
Electron exposure ($e^-/Å^2$)	40
Defocus range (µm)	–0.8 to –1.6
Pixel size (Å)	0.925
Symmetry imposed	No
Initial helical segments (no.)	126,987
Final helical segments (no.)	104,748

PuuE D-loop tube preincubated on ice to unwind the helical structures

	#1 C_3 tube (EMD-60620)	#2 C_4 tube (EMD-60621)	#3 C_5 tube (EMD-60622)	#4 C_6 tube (EMD-60623)
**Data collection and processing**				
Magnification	150,000			
Voltage (kV)	200			
Electron exposure ($e^-/Å^2$)	40			
Defocus range (µm)	–0.8 to –1.6			
Pixel size (Å)	0.925			
Symmetry imposed	C_3 helical	C_4 helical	C_5 helical	C_6 helical
Initial helical segments (no.)	397,778	397,778	397,778	397,778
Final helical segments (no.)	2,675	3,262	2,998	1,291
Map resolution (Å)	9.7	14.6	18.2	26.0
FSC threshold	0.143	0.143	0.143	0.143

Supplementary Table 2. Cryo-EM data collection, refinement, and validation statistics.
