# Supplementary material for: Protein design of two-component tubular assemblies similar to cytoskeletons
Source: Nat Commun. 2025 Jul 22;16:6738. doi: 10.1038/s41467-025-62076-3 (PMC12283931; doi:10.1038/s41467-025-62076-3)
Supplement: Supplementary file 2 — Description of Additional Supplementary Files [file 41467_2025_62076_MOESM2_ESM.pdf]

Description of Additional Supplementary Files

File name: Supplementary Data 1

Description: Primer sequences used in this study.

File name: Supplementary Movie 1

Description: Structural diversity of PuuE tube. Maps with tube structures obtained by 3D classification were transitioned linearly using the *vop morph* function in the ChimeraX software to show their structural differences. Different diameters of PuuE tubes with C_4, C_5, and C_6 symmetry were observed from cryo-EM analysis, suggesting contraction of the PuuE tube.

File name: Supplementary Movie 2

Description: Fitting of PuuE-p based on AF2 predictions into the 3D reconstructed model of PuuE tubes with C_4 symmetry. The results from the fitting analysis indicate that the PuuE-p model does not align well with units situated inside the tube structure; however, it aligns more appropriately with the units on the exterior. The 6xHis-TEVcs region of the PuuE-p model is not shown to increase visibility.

File name: Supplementary Movie 3

Description: Dynamic flexibility of PuuE tube. The structural flexibility of PuuE tubes was observed in real-time using TIRFM over a period of 8 seconds. Field of view: 87 µm x 87 µm.

File name: Supplementary Movie 4

Description: Fitting of PuuE-p based on AF2 predictions into the 3D reconstructed model of single PuuE D-loop tubes with C_3 symmetry. The results from the fitting analysis indicate that the PuuE-p model does not align well with units situated inside the tube structure; however, it aligns more appropriately with the units on the exterior. The 6xHis-TEVcs region of the PuuE-p model is not shown to increase visibility.
